# 气相色谱-串联质谱法测定食用油中角鲨烯和氧化角鲨烯及角鲨烯热稳定性评价

**DOI:** 10.3724/SP.J.1123.2024.05031

**Published:** 2025-07-08

**Authors:** Gengpeng XIAO, Dandan LIAO, Guisheng LI, Xiang LUO, Lu YUAN

**Affiliations:** 江西省检验检测认证总院，检测认证技术发展研究院，江西 南昌 330029; Development Research Institute of Testing and Certification Technology，Jiangxi General Institute of Testing and Certification，Nanchang 330029，China

**Keywords:** QuEChERS, 气相色谱-串联质谱, 角鲨烯, 氧化角鲨烯, QuEChERS, gas chromatography-tandem mass spectrometry （GC-MS/MS）, squalene, oxidized squalene

## Abstract

基于QuEChERS-气相色谱-串联质谱建立了食用油中角鲨烯和氧化角鲨烯的含量测定方法。采用正己烷提取食用油样品，使用乙二胺-*N*-丙基硅烷化硅胶（PSA）与硅胶（CNW BOND Si）的混合吸附剂进行净化处理。通过TG-5ms色谱柱（30 m×0.25 mm×0.25 μm）进行分离，并以角鲨烷作为内标物，在选择反应监测（SRM）模式下应用内标法进行定量分析。实验对色谱柱和吸附剂的选择分别进行了优化。在优化的实验条件下经方法学验证表明，角鲨烯和氧化角鲨烯分别在0.03~0.4 mg/L和0.29~3.80 mg/L范围内线性关系良好，相关系数均≥0.992。角鲨烯和氧化角鲨烯的检出限（LOD，*S/N*=3）分别为0.4 mg/kg和4.0 mg/kg，定量限（LOQ，*S*/*N*=10）分别为1.2 mg/kg和12 mg/kg。在3种不同类型的食用油中分别进行加标回收试验，在低、中、高3个加标水平下，角鲨烯和氧化角鲨烯的回收率分别为81.9%~102.5%和89.4%~116.1%，相对标准偏差（RSD， *n*=6）分别为3.5%~6.8%和3.2%~7.4%。该方法具有操作简便、稳定可靠、LOD低等优势，适用于食用油中角鲨烯和氧化角鲨烯的检测。利用该方法评价了花生油中角鲨烯的热稳定性，结果发现，当温度低于120 ℃时，角鲨烯未发生转化为氧化角鲨烯的现象；然而，当温度超过120 ℃后，角鲨烯的峰面积出现断崖式下降，且可明显检测到氧化角鲨烯的生成。利用该方法对菜籽油、花生油、大豆油和玉米油4类食用油进行检测，结果显示，所有样品均检出角鲨烯，而氧化角鲨烯未在任何样品中检出。该方法旨在为食用油的储存管理以及品质快速鉴别提供新的思路。

## Abstract (en)

The quality and safety of edible oils have frequently been compromised in recent years， seriously threatening consumer’s legitimate rights and health. Hence， establishing methods for determining the quality of edible oils based on their endogenous components is greatly significant. Squalene is widely present in various oils； hence， studying the thermal stability of squalene in edible oils is expected to provide a new storage-management model and a method for rapidly determining oil quality. A method for determining squalene and oxidized squalene in edible oils was established based on QuEChERS-gas chromatography-tandem mass spectrometry. Edible oil samples were extracted by *n*-hexane and purified using a mixed adsorbent consisting of ethylenediamine-*n*-propyl silanized silica gel （PSA） and silica gel （CNW BOND Si）. Separation was used a TG-5ms column （30 m×0.25 mm×0.25 μm） and the squalene was used as an internal standard for quantitative analysis in selective reaction monitoring （SRM） mode. The chromatography column and adsorbent were judiciously optimized. Methodological verification revealed good linear relationships for squalene and oxidized squalene in the ranges of 0.03–0.4 and 0.29–3.80 mg/L， respectively， with correlation coefficients （*r*）≥0.992 under the optimized experimental conditions. Limits of detection （LODs， *S/N*=3） of 0.4 and 4.0 mg/kg were obtained for squalene and oxidized squalene， respectively， with corresponding limits of quantification （LOQs， *S/N*=10） of 1.2 and 12 mg/kg， respectively. Spiked recovery experiments were conducted at low， medium， and high spiked levels using three different oils， the average recoveries of squalene and oxidized squalene were 81.9%‒102.5% and 89.4%‒116.1%， respectively， with relative standard deviations （RSDs， *n*=6） of 3.5%‒6.8% and 3.2%‒7.4%， respectively. The developed method has the advantages of operational simplicity， stability， reliability， low LODs， and is suitable for detecting squalene and oxidized squalene in edible oils. The developed method was used to evaluate the thermal stability of squalene in peanut oil. The results showed that when the temperature was lower than 120 ℃， squalene did not undergo the phenomenon of conversion to oxidized squalene. However， when the temperature exceeds 120 ℃， the peak area of squalene shows a cliff-like decline， and the formation of oxidized squalene can be clearly detected. This method was used to test rapeseed oil， peanut oil， soybean oil and corn oil. The results showed that squalene was detected in all samples， while oxidized squalene was not detected in any samples. This method aims to provide new ideas for the storage management and rapid quality identification of edible oil.

角鲨烯（squalene），亦称鲨烯，是一种自然存在于动植物油脂中的多不饱和烃类物质。由于其分子结构中包含六个非共轭双键，角鲨烯展现出多种生物活性，包括促进新陈代谢、增强机体免疫力、抗肿瘤、抗氧化以及活化细胞等功能。因此，它在食品、医药及化妆品等多个领域得到了广泛应用^［1-3］^。最初，角鲨烯被人们在深海鲨鱼肝油中发现。随着研究的逐步深入，科学家们还发现橄榄油、油茶籽油、菜籽油、花生油、大豆油、玉米油以及葵花籽油等多种植物油中均含有一定量的角鲨烯^［4，5］^。然而，研究表明，角鲨烯在遇到光照、高温、紫外线辐射、环境氧化剂以及皮肤微生物群等多种应激因素时，容易发生氧化和分解反应。例如，Nakagawa等^［6］^研究发现，在阳光暴露下，人体皮脂中的角鲨烯会生成6种角鲨烯单羟基过氧化物异构体。目前，关于食用油中角鲨烯稳定性的研究主要聚焦于光照应激条件，包括正常光照与紫外线照射。黎斌等^［7］^发现，在正常光照和室温下放置9天后，橄榄油中角鲨烯的降解速度逐渐趋于稳定。张欢等^［8］^考察了大豆油中角鲨烯在紫外光辐射下的稳定性，并鉴定了其光损耗产物，发现角鲨烯损耗后产生了两种大分子物质：一种是羟基衍生物，另一种是环氧衍生物。现有文献多聚焦于光照应激条件下角鲨烯的稳定性研究，但相关实验普遍周期较长。此类研究虽对油品储存具有指导意义，却难以有效鉴别油品质量。在煎炸用油和地沟油等油脂类物质的重复利用过程中，均需采用高温处理工艺，但目前缺乏对高温处理条件下角鲨烯热稳定性的研究。探究角鲨烯在高温下转化为氧化角鲨烯的规律，有助于将氧化角鲨烯作为特征标志物，用于食用油品质的鉴定。因此，建立一种专属性强、灵敏度高的分析方法，用于检测食用油中的角鲨烯和氧化角鲨烯，并研究角鲨烯的热稳定性，对于油品质量鉴别具有重要意义。

目前，文献中报道的角鲨烯检测方法主要包括液相色谱法（HPLC）^［9，10］^、气相色谱-氢火焰离子化法（GC-FID）^［11-14］^、气相色谱-质谱法（GC-MS）^［15-19］^以及气相色谱-串联质谱法（GC-MS/MS）^［20］^。在这些方法中，HPLC和GC-FID在复杂基质中的定性能力较弱，且灵敏度不高，因此在痕量化合物的检测中并不具备明显优势。相较于GC和HPLC，GC-MS虽在灵敏度和特异性上有所提升，但其提供的结构碎片离子信息有限，导致定性准确度仍有欠缺。GC-MS/MS通过采用选择反应监测（SRM）模式来选择特定的离子对，不仅增强了定性准确度，还提高了灵敏度，特别适用于基质干扰严重情况下的痕量化合物分析。此外，目前报道的角鲨烯前处理方法主要包括皂化-萃取法和固相萃取法，但这些方法存在操作步骤繁琐、耗时长以及有机溶剂消耗量大等缺点^［21］^。相比之下，QuEChERS作为近年来新兴的快速样品前处理技术，因其操作简便、分析速度快、溶剂消耗少且高效等优势，已被广泛应用于农兽药残留检测领域。截至目前，尚未有相关文献报道关于氧化角鲨烯的定量检测方法。

综上，本文采用QuEChERS-GC-MS/MS，建立了一种操作简便、稳定性强、灵敏度高的方法，用于同时测定食用油中的角鲨烯和氧化角鲨烯。利用该方法，我们不仅评估了食用油中角鲨烯的热稳定性，还进行了实际样品的测定。通过探究角鲨烯在高温条件下转化为氧化角鲨烯的规律，期望能以氧化角鲨烯为特征标志物，实现对食用油储存状态及品质的快速鉴别。

## 1 实验部分

### 1.1 仪器、试剂与材料

TRACE 1300-TSQ 9000气相色谱-串联质谱联用仪（美国Thermo Fisher Scientific公司）；VORTEX 3型涡旋混合器（德国IKA公司）；HC-2062高速离心机（科大创新公司）；Mettler Toledo AL204电子分析天平（瑞士Mettler Toledo公司）。

角鲨烯（含量≥98%）和角鲨烷（含量≥99%）均购自上海麦克林生化科技有限公司；氧化角鲨烯（含量≥92%）购自美国Sigma-Aldrich公司；正己烷（色谱纯）、乙二胺-*N*-丙基硅烷化硅胶（PSA）、十八烷基键合硅胶（CNW BOND HC-C_18_，C_18_）及硅胶（CNW BOND Si）均购自上海安谱实验科技股份有限公司。

实验中用到的食用油购买于本地市场。

### 1.2 标准溶液的配制

#### 1.2.1 储备液的配制

准确称取0.01 g（精确到0.000 1 g）角鲨烯，置于100 mL容量瓶中，用正己烷溶解并定容，摇匀后即得质量浓度为100 mg/L的角鲨烯标准储备液。

准确称取0.01 g（精确到0.000 1 g）氧化角鲨烯，置于100 mL容量瓶中，用正己烷溶解并定容，摇匀后即得质量浓度为100 mg/L氧化角鲨烯标准储备液。

准确称取0.01 g（精确到0.000 1 g）内标角鲨烷，置于250 mL容量瓶中，用正己烷溶解并定容，摇匀后即得质量浓度为40 mg/L的内标储备液。

#### 1.2.2 混合标准工作溶液的配制

分别移取不同体积的角鲨烯标准储备液和氧化角鲨烯标准储备液，用正己烷逐级混合稀释，随后分别加入相同体积的内标储备液，配制成系列质量浓度的混合标准工作溶液。其中，内标的质量浓度为0.1 mg/L，角鲨烯的质量浓度分别为0.03、0.05、0.1、0.2、0.4 mg/L，氧化角鲨烯的质量浓度分别为0.29、0.48、0.95、1.9、3.8 mg/L。

#### 1.2.3 基质匹配混合标准溶液的配制

分别移取不同体积的角鲨烯标准储备液和氧化角鲨烯标准储备液，用基质提取液逐级混合稀释后，分别加入相同体积的内标储备液，配制成系列质量浓度的基质匹配混合标准溶液。其中，内标的质量浓度为0.1 mg/L，角鲨烯的质量浓度分别为0.03、0.05、0.1、0.2、0.4 mg/L，氧化角鲨烯的质量浓度分别为0.29、0.48、0.95、1.9、3.8 mg/L。

### 1.3 样品前处理

准确称取0.2 g（精确到0.001 g）食用油样品于10 mL比色管中，用正己烷溶解后，涡旋分散均匀。移取3 mL样品提取液，加入到预先混合有0.3 g PSA和0.3 g CNW BOND Si的具塞试管中，涡旋振荡5 min，在4 000 r/min下离心10 min。将上清液过0.22 μm滤膜，供GC-MS/MS测定。

### 1.4 仪器条件

#### 1.4.1 气相色谱条件

色谱柱：TG-5ms型石英毛细管柱（30 m×0.25 mm×0.25 μm）；载气：氦气（纯度>99.999%）；柱流量：1.2 mL/min，分流进样，分流比为1∶5；进样体积：1 μL；进样口温度：270 ℃。柱温箱温度采用程序升温控制，先在120 ℃保持1 min，随后以30 ℃/min速率升温至280 ℃，并在该温度下保持15 min。

#### 1.4.2 质谱条件

离子源：电子轰击电离（EI）源，电子轰击能量为70 eV；传输线温度：280 ℃；离子源温度：280 ℃；监测模式：SRM模式。角鲨烯、氧化角鲨烯及内标的保留时间、定量和定性离子列于表1中。

**表1:** 角鲨烯、氧化角鲨烯及内标的保留时间和质谱参数

Compound	*t*_R_/min	Ion pairs （*m/z*）	CEs/eV
Squalene	9.61	81.1/79.1^*^， 69.1/41.1， 69.1/39.1， 121.1/93.1	10， 10， 15， 10
Oxidized squalene	10.62	81.1/79.1^*^， 69.1/41.1， 69.1/39.1， 81.1/41.1	10， 10， 15， 15
	10.85	81.1/79.1^*^， 69.1/41.1， 69.1/39.1， 81.1/41.1	10， 10， 15， 15
	11.04	81.1/79.1^*^， 69.1/41.1， 69.1/39.1， 81.1/41.1	10， 10， 15， 15
	11.46	81.1/79.1^*^， 69.1/41.1， 69.1/39.1， 81.1/41.1	10， 10， 15， 15
Squalane （IS）	8.42	71.1/43.1^*^， 85.1/43.1， 57.1/41.1	5， 10， 5

CEs： collision energies； * quantitative ion.

## 2 结果与讨论

### 2.1 仪器条件的优化

角鲨烯和氧化角鲨烯分别为弱极性和中等弱极性化合物，在实验方法探索初期，比较了两种具有中等偏弱极性的色谱柱（TG-1701ms石英毛细管柱（30 m×0.25 mm×0.25 μm）和TG-5ms石英毛细管柱（30 m×0.25 mm×0.25 μm））对目标化合物的分离效果，其中TG-1701ms色谱柱的极性高于TG-5ms色谱柱。结果表明，两种色谱柱均能够分离目标化合物，但使用TG-5ms色谱柱时，目标化合物的色谱峰形更加尖锐，且该色谱柱的适用温度较高，有利于高沸点化合物的洗脱。因此，本研究最终选择TG-5ms色谱柱作为分离柱。

用正己烷配制角鲨烯、氧化角鲨烯和内标质量浓度均为10 mg/L的混合标准溶液，在EI源条件下进行全扫描分析，通过美国国家标准与技术研究院谱库（NIST）检索确认各目标化合物的分子结构及保留时间。角鲨烯、氧化角鲨烯和内标角鲨烷的总离子流色谱图（TIC）和SRM色谱图见图1。结果显示，氧化角鲨烯呈现4个异构体色谱峰（图1a和1c）。结合试剂生产厂家提供的分析证书（COA）及文献数据，推测其异构体结构包含顺式/反式角鲨烯单羟基氧化物和顺式/反式环氧角鲨烯；定量分析采用各异构体峰面积加和的方式进行计算^［22］^。针对各目标化合物，分别选取响应强度较高且*m/z*较大的离子作为母离子，再通过二次碰撞进行子离子扫描，同样选取响应强度较高且*m/z*较大的离子作为子离子，并对碰撞能进行优化。经优化后确定各目标化合物的定量离子对、定性离子对及碰撞能参数，具体数值详见表1。

**图1:**
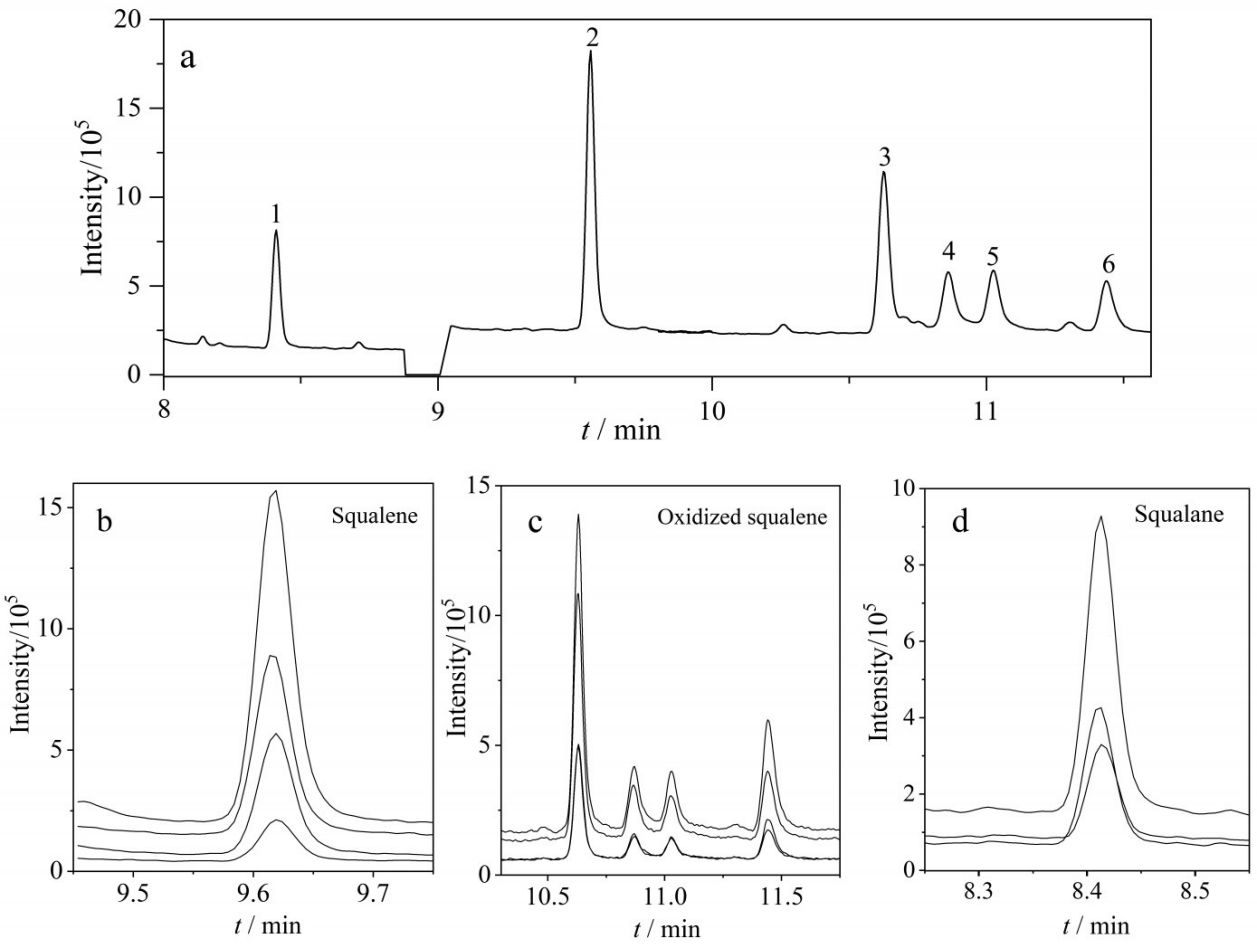
角鲨烯、氧化角鲨烯和内标的（a）总离子流色谱图及（b~d）SRM色谱图

### 2.2 吸附剂种类及用量的优化

食用油样品种类繁多且基质成分复杂，既含有高沸点组分，又存在大量大分子干扰物质，其中高沸点物质容易在进样口和柱前端发生聚集，导致测定结果异常。因此，需采用合适的吸附剂来去除基质样品中的这些杂质。本研究考察了3种常用的固相萃取吸附剂（C_18_（非极性）、PSA（极性）、CNW BOND Si（极性））对目标化合物的净化效果。实验发现，C_18_对目标化合物有明显的吸附作用，会导致目标化合物损失；然而，未见PSA和CNW BOND Si对目标化合物有明显的吸附，这是因为目标化合物为弱极性化合物，而PSA和CNW BOND Si属于极性吸附剂，对其吸附作用小。考虑到食用油中既含有脂肪酸甘油酯和磷脂等中等极性化合物，也可能含有游离脂肪酸、甘油一酸酯、甘油二酸酯及多种氧化产物等强极性化合物，为了使角鲨烯和氧化角鲨烯净化完全，本实验最终选用PSA和CNW BOND Si的混合吸附剂来对目标化合物进行净化。此外，实验还进一步考察了PSA和CNW BOND Si的用量（0.1、0.2、0.3、0.4、0.5 g）对目标化合物回收率的影响，结果如图2所示。随着PSA和CNW BOND Si用量的增加，角鲨烯和氧化角鲨烯的回收率变化不大；但总体来看，当二者的用量均为0.3 g时，角鲨烯和氧化角鲨烯的回收率最高。为了实现目标化合物的充分净化并避免浪费，本文使用0.3 g PSA和0.3 g CNW BOND Si混合吸附剂对食用油样品进行净化。

**图2:**
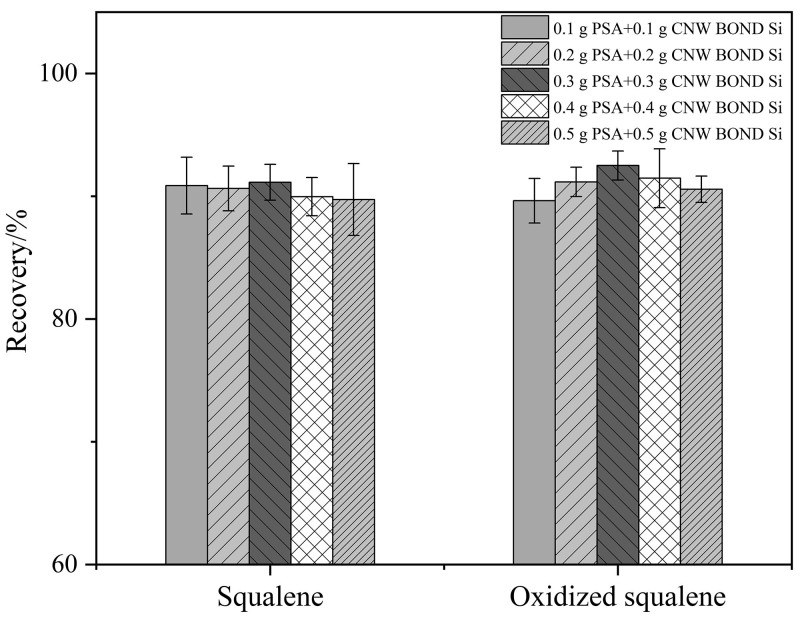
混合吸附剂用量对目标化合物回收率的影响（*n*=3）

### 2.3 基质效应

基质效应（matrix effect，ME）是指在质谱分析中，样品基质对目标化合物的电离产生影响，进而影响离子化效率，导致信号响应受到抑制或增强的现象，这种基质效应会影响测定结果的准确性。本文以角鲨烯含量较低的菜籽油为研究对象来考察基质效应，按1.2节方法，分别配制基质匹配混合标准溶液和溶剂混合标准工作溶液，进样测定并绘制相应的标准曲线（其中基质匹配标准曲线是在扣除样品本底峰面积后绘制的）。通过计算二者线性回归方程的斜率比来评估基质效应，即ME=*k*_基质_/*k*_溶剂_。一般来说，当ME>1.15时表现为基质增强效应，当0.85≤ME≤1.15时可视为不存在基质效应，当ME<0.85时表现为基质抑制效应^［23］^。通过实验计算得到角鲨烯和氧化角鲨烯的ME分别为0.86和1.05，表明本方法几乎不存在基质效应。因此，为在简化分析流程的同时保证定量准确性，本文采用溶剂混合标准工作溶液进行定量分析。

### 2.4 方法学评价

#### 2.4.1 相关系数、检出限和定量限

将溶剂混合标准工作溶液在优化的仪器条件下进样分析，以目标化合物与内标质量浓度的比值为横坐标（*x*），目标化合物与内标峰面积的比值为纵坐标（*y*），采用最小二乘法进行线性回归分析，得到各目标化合物的线性方程和相关系数，结果见表2。结果表明，角鲨烯和氧化角鲨烯在各自的线性范围内具有良好的线性关系，相关系数（*r*）均≥0.992。以空白样品的3倍信噪比（*S/N=*3）和*S/N=*10来计算检出限（LOD）和定量限（LOQ），结果表明，角鲨烯的LOD和LOQ分别为0.4 mg/kg和1.2 mg/kg，氧化角鲨烯的LOD和LOQ分别为4.0 mg/kg和 12 mg/kg。

**表2:** 角鲨烯和氧化角鲨烯的线性范围、线性方程、相关系数、检出限及定量限

Compound	Linear range/（mg/L）	Linear equation	*r*	LOD/（mg/kg）	LOQ/（mg/kg）
Squalene	0.03-0.4	*y*=2.667*x*+0.016	0.996	0.4	1.2
Oxidized squalene	0.29-3.80	*y*=0.928*x*-0.085	0.992	4.0	12

*y：* peak area ratio of target compound to internal standard； *x：* mass concentration ratio of target compound to internal standard.

#### 2.4.2 回收率和精密度

选择3种不同类型（大豆油、菜籽油、玉米油）的食用油样品，分别加入低、中、高3个水平的角鲨烯和氧化角鲨烯标准储备液，并加入相应体积的内标储备液，每个加标水平平行制备6份。其中，角鲨烯的3个加标水平分别为上述3种样品本底值（13.8、14.1、50.4 mg/kg）的1、2、4倍，氧化角鲨烯的3个加标水平分别为15、60、150 mg/kg。按照优化的实验条件进行样品前处理和进样分析，计算平均回收率和相对标准偏差（RSD），结果见表3。在低、中、高3个加标水平下，角鲨烯和氧化角鲨烯在3种食用油中的平均回收率分别为81.9%~102.5%和89.4%~116.1%，RSD分别为3.5%~6.8%和3.2%~7.4%。实验结果表明，所建方法具有良好的准确度和精密度。

**表3:** 3种食用油中角鲨烯和氧化角鲨烯的平均回收率和相对标准偏差（*n*=6）

Compound	Soybean oil			Rapeseed oil			Corn oil		
	Spiked level/ （mg/kg）	Recovery/%	RSD/%	Spiked level/ （mg/kg）	Recovery/%	RSD/%	Spiked level/ （mg/kg）	Recovery/%	RSD/%
Squalene	13.8	86.7	6.8	14.1	84.7	5.1	50.4	82.3	4.6
	27.6	88.1	6.5	28.2	84.5	4.7	100.8	81.9	3.5
	55.2	92.8	5.8	56.4	99.4	5.5	201.6	102.5	6.7
Oxidized squalene	15	89.4	7.2	15	93.2	7.0	15	94.2	6.7
	60	91.2	6.9	60	105.4	7.1	60	112.4	7.4
	150	116.1	4.6	150	94.4	4.0	150	103.7	3.2

### 2.5 热稳定性评价

本研究通过比较不同温度处理条件下样品中角鲨烯与氧化角鲨烯的色谱峰面积变化，评估角鲨烯的热稳定性。选择角鲨烯含量较高的花生油作为热稳定性研究对象。将花生油分别在25、80、100、120、150、180、200 ℃条件下放置3 h，考察内源性角鲨烯的热稳定性，结果如图3a所示。随着温度升高，角鲨烯的峰面积逐渐降低，在升温初期（温度低于120 ℃），角鲨烯的峰面积变化较小，但当温度高于120 ℃后，角鲨烯的峰面积出现断崖式下降；当升温至200 ℃时，角鲨烯的峰面积仅为室温（25 ℃）条件下的17.7%。此外，实验还研究了角鲨烯向氧化角鲨烯转化的规律。结果表明，当温度低于120 ℃时，角鲨烯未发生转化为氧化角鲨烯的现象，这可能是因为环境温度较低，转化率低。然而，当温度超过120 ℃后，可明显检测到氧化角鲨烯的生成，说明发生了角鲨烯向氧化角鲨烯转化的现象；并且，氧化角鲨烯的含量随温度的升高而增加，在温度超过150 ℃后，氧化角鲨烯的含量趋于平稳。

**图3:**
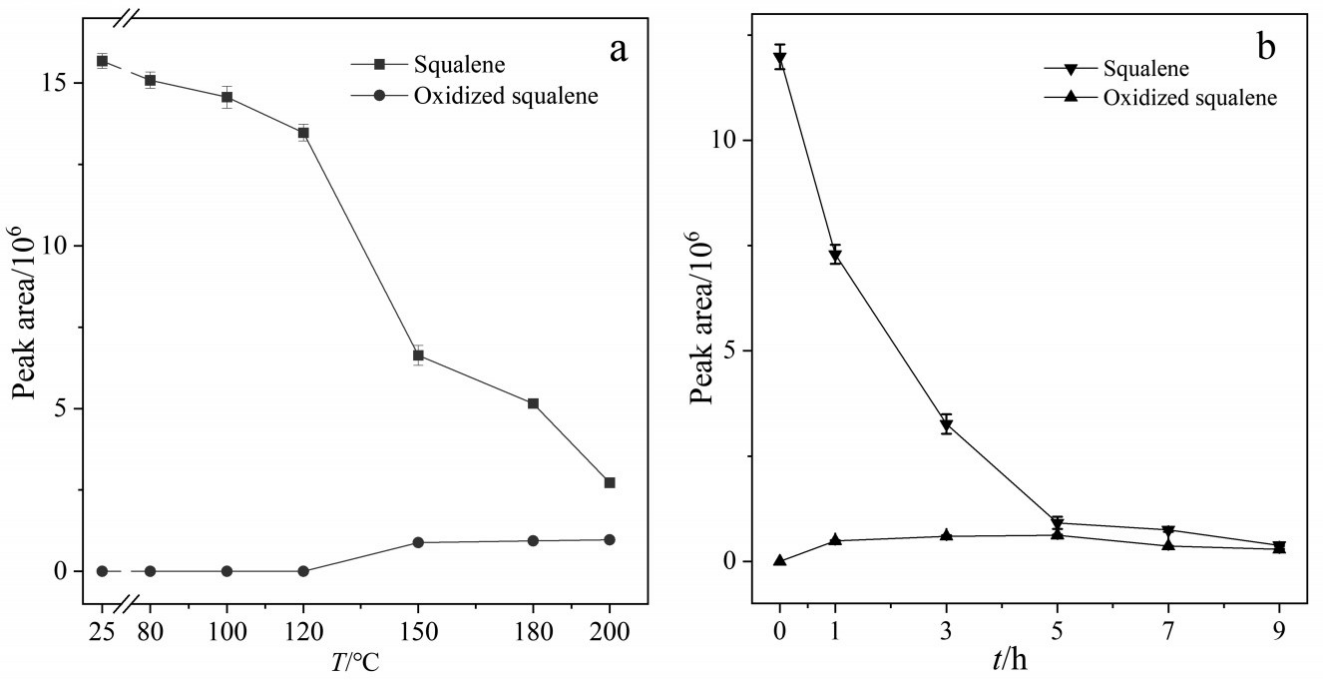
角鲨烯和氧化角鲨烯的峰面积随（a）加热温度和（b）加热（200 ℃）时间的变化（*n*=3）

最后，实验考察了花生油分别在200 ℃下高温处理0、1、3、5、7、9 h后内源性角鲨烯的含量变化（图3b）。结果表明，随着高温处理时间的延长，角鲨烯的峰面积逐渐降低，且在前5 h内降解速率最快，5 h后角鲨烯的峰面积趋于稳定。同时发现，氧化角鲨烯的含量随高温处理时间的延长而逐渐增加，并在5 h达到峰值后缓慢下降，这可能是由于氧化角鲨烯在持续高温条件下会进一步环化生成萜类化合物。此外，实验还发现角鲨烯转化为氧化角鲨烯的物质的量比并非1∶1。基于这一现象，我们对高温处理后的角鲨烯原料进行了成分鉴别，结果表明，除生成氧化角鲨烯外，角鲨烯在高温下还会产生大量的正己烷不溶物。

### 2.6 实际样品检测

采用本文所建立的方法对从市场上随机采购的菜籽油、花生油、大豆油和玉米油4类食用油进行检测，每类各取5份样品，共20份。当样液中待测组分含量超出标准工作曲线线性范围时，需对样液进行适当稀释，确保其进样浓度在线性范围之内。检测结果显示，所有样品均检出角鲨烯，而氧化角鲨烯未在任何样品中检出。其中，花生油中的角鲨烯含量最高（143.1~479.1 mg/kg），菜籽油中的角鲨烯含量为14.1~27.1 mg/kg，大豆油中的角鲨烯含量为13.8~64.4 mg/kg，玉米油中的角鲨烯含量为50.4~134.4 mg/kg。对不同原料品种的角鲨烯含量分析表明，其含量存在差异，但无明确规律性分布。这可能是因为角鲨烯的含量不仅与原料品种有关，还与原料产地、加工方式、储存环境和温度及包装材料等有关。

## 3 结论

基于QuEChERS-气相色谱-串联质谱，本文建立了测定食用油中角鲨烯和氧化角鲨烯含量的分析方法。该方法具有操作简便、稳定可靠、LOD低等优势，适用于食用油中角鲨烯和氧化角鲨烯的检测。通过热稳定性评价实验发现，在温度低于120 ℃时，角鲨烯未发生转化为氧化角鲨烯的现象，而当温度超过120 ℃后，可明显观察到角鲨烯转化为氧化角鲨烯。此外，在高温（200 ℃）处理长达5 h后，除生成氧化角鲨烯外，角鲨烯还会产生大量的正己烷不溶物。本方法的建立有助于为后续深入研究食用油中的角鲨烯提供技术支撑，并为食用油的储存管理以及品质快速鉴别提供新的思路。
